# Bone health and HIV in resource-limited settings: a scoping review

**DOI:** 10.1097/COH.0000000000000274

**Published:** 2019-01-14

**Authors:** Flavia Kiweewa Matovu, Lalita Wattanachanya, Mags Beksinska, John M. Pettifor, Kiat Ruxrungtham

**Affiliations:** a Makerere University-Johns Hopkins University (MU-JHU) Research Collaboration; b Department of Epidemiology and Biostatistics, Makerere University School of Public Health, Kampala, Uganda; c School of Public Health, University of the Witwatersrand, Johannesburg, South Africa; d Division of Endocrinology and Metabolism, Department of Medicine, and Hormonal and Metabolic Disorders Research Unit, Faculty of Medicine, Chulalongkorn University; e Excellence Center for Diabetes, Hormone, and Metabolism, King Chulalongkorn Memorial Hospital, Thai Red Cross Society, Bangkok, Thailand; f Maternal, Adolescent and Child Health (MatCH) Research, University of the Witwatersrand, Faculty of Health Sciences, Department of Obstetrics and Gynaecology; g MRC/Wits Developmental Pathways for Health Research Unit, Faculty of Health Sciences, University of the Witwatersrand, Johannesburg, South Africa; h Department of Medicine, Faculty of Medicine, Chulalongkorn University; i HIV-NAT, Thai Red Cross AIDS Research Center, Thai Red Cross Society, Bangkok, Thailand; ∗ Flavia Kiweewa Matovu and Lalita Wattanachanya contributed equally to the writing of this article.

**Keywords:** antiretroviral therapy, bone mineral density, HIV, resource-limited settings, tenofovir, vitamin D

## Abstract

**Purpose of review:**

Sub-Saharan Africa and other resource-limited settings (RLS) bear the greatest burden of the HIV epidemic globally. Advantageously, the expanding access to antiretroviral therapy (ART) has resulted in increased survival of HIV individuals in the last 2 decades. Data from resource rich settings provide evidence of increased risk of comorbid conditions such as osteoporosis and fragility fractures among HIV-infected populations. We provide the first review of published and presented data synthesizing the current state of knowledge on bone health and HIV in RLS.

**Recent findings:**

With few exceptions, we found a high prevalence of low bone mineral density (BMD) and hypovitaminosis D among HIV-infected populations in both RLS and resource rich settings. Although most recognized risk factors for bone loss are similar across settings, in certain RLS there is a high prevalence of both non-HIV-specific risk factors and HIV-specific risk factors, including advanced HIV disease and widespread use of ART, including tenofovir disoproxil fumarate, a non-BMD sparing ART. Of great concern, we neither found published data on the effect of tenofovir disoproxil fumarate initiation on BMD, nor any data on incidence and prevalence of fractures among HIV-infected populations in RLS.

**Summary:**

To date, the prevalence and squeal of metabolic bone diseases in RLS are poorly described. This review highlights important gaps in our knowledge about HIV-associated bone health comorbidities in RLS. This creates an urgent need for targeted research that can inform HIV care and management guidelines in RLS.

**Video abstract:**

http://links.lww.com/COHA/A9.

## INTRODUCTION

Resource-limited settings (RLS) that constitute low- and middle-income countries [1] continue to bear the greatest burden of the HIV epidemic globally [2]. Advantageously, the expanding access to highly active antiretroviral therapy has resulted in dramatically increased survival of HIV-infected individuals in the last 2 decades [3]. With more HIV-infected individuals living longer, it is expected that medical comorbidities such as osteoporosis and fragility fractures will increase. Data from developed countries estimate that up to two-thirds of HIV-infected antiretroviral therapy (ART)-treated and ART-naïve individuals exhibit osteopenia or osteoporosis at the time of low bone mineral density (BMD) diagnosis, with those on ART at increased risk [4]. Importantly, studies in resource rich settings (RRS) are reporting increased evidence of fracture rates in the HIV-infected population, with fracture rates 30–70% higher than those among matched uninfected controls [5,6^▪^,7^▪^,^8^,^9^]. Fragility fractures are associated with significant loss of physical function, independence, and quality of life [10], as well as an increased risk of short-term and long-term mortality [11–13]. These data call for more strategic clinical management of HIV individuals that includes prevention or minimization of long-term metabolic complications of HIV infection and its treatment in addition to treating opportunistic infections. In this review, we summarize recently published and presented studies that inform the discussion on bone health among HIV-infected persons in RLS. We highlight the epidemiology of HIV and bone loss in RLS, and among special populations, including HIV-infected young women and perinatally infected adolescents. We focus on three main areas of interest in HIV metabolic bone disease in RLS: effects of HIV and ART, vitamin D insufficiency and other risk factors for bone loss, and fracture risk assessment. We further identify important gaps in research and clinical management as well as make recommendations for future research priorities that would help address these HIV-related, bone health comorbidities in RLS.

### Bone mass and HIV in resource-limited settings

The World Health Organization has categorized low BMD into osteopenia and osteoporosis. In postmenopausal women and men, 50 years and above, osteoporosis is defined as a T-score at or below 2.5 SD whereas osteopenia is defined as a T-score between 1 and 2.5 SD below the young adult mean value. Premenopausal women, men below 50 years or children who have a BMD Z-score at or below 2.0 of the age and sex-matched population are classified as having low bone mass. [14]. In the general population, a decline in BMD, assessed by dual-energy X-ray absorptiometry (DXA), is associated with an increased risk of subsequent fractures [15]. Data from RRS consistently show that HIV infection is associated with low BMD and increased fracture risk [5,6^▪^,7^▪^,^8^,^9^]. A meta-analytic review of 11 studies by Brown *et al.* involving 884 HIV-infected individuals and 654 controls estimated the prevalence of low BMD among HIV-infected individuals to be as high as 67%, 15% of whom had osteoporosis. The magnitude of low BMD was 6.4 times greater and that of osteoporosis 3.7 times greater than in HIV-uninfected controls [4]. Further, in a recent meta-analysis, fracture risk was 1.35-fold higher in HIV-positive compared to HIV-negative controls [7^▪^]. Although underlying mechanisms leading to reduced BMD in HIV-infected persons are still unclear, they are believed to be multifactorial and include both traditional and HIV-specific risk factors [4,16–25]. Owing to physiological, psychological, and lifestyle factors, HIV-infected persons are likely to have many of the traditional risk factors for low BMD such as physical inactivity, low body weight, nutritional deficiencies (including inadequate calcium and vitamin D intake), depression, smoking, heavy alcohol use, oligo-/amenorrhoea, and hypogonadism [26–35]. Among the nontraditional causes, a direct effect of HIV and its treatment have been most often quoted; chronic inflammation induced by HIV may impact bone metabolism [36–39]. In addition, ART significantly contributes to bone loss among HIV-infected persons [40]. Among individuals on ART, studies in RRS consistently report a 2–6% decline in BMD over the first few years after treatment initiation [25,41], regardless of ART choice [26].

**FIGURE 1:**
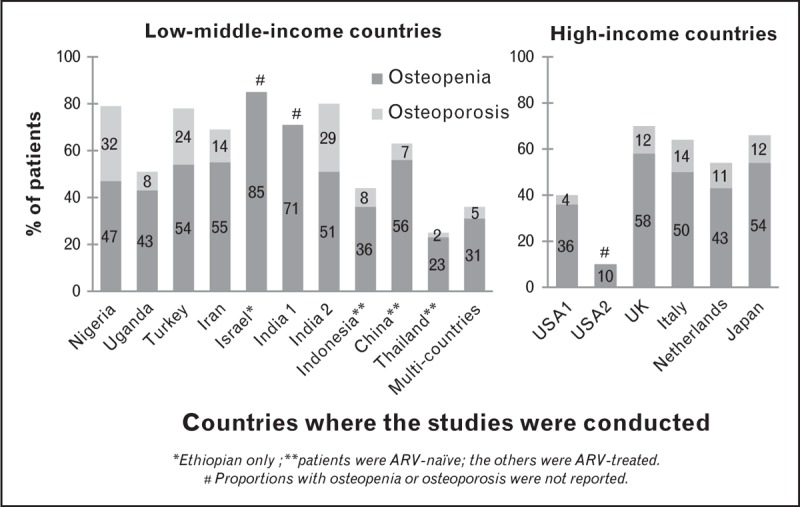
Proportion of HIV-infected patients with low bone mineral density, osteopenia, and osteoporosis. Overlapping prevalence of osteopenia and osteoporosis in low-/low–middle-income countries and high-income countries was found, with a generally higher prevalence of low bone mineral density in low- to middle-income countries overall compared to high-income countries.

In RLS with a disproportionately high burden of HIV and background nutritional deficiencies [42], known risk factors for low BMD remain similar to those in RRS [25,43,44]. However, some of these risk factors such as low BMI, malnutrition, advanced disease, longer duration since HIV diagnosis and higher HIV viral load are more common in HIV-infected populations in RLS [45^▪^,^46^,47^▪^,^48^]. These risk factors coupled with more widespread use of non-BMD sparing ART-like tenofovir disoproxil fumarate (TDF) and efavirenz (EFV) make the extremely high prevalence of low BMD in some RLS almost inevitable. Unfortunately, data on BMD among HIV-infected individuals are currently scanty and subject to methodological concerns such as cross-sectional design, lack of appropriate control groups, and local BMD reference data. The majority of the studies did not use local noninfected controls for comparison; the United States National Health and Nutrition Examination Survey reference data being used instead and comparisons were not adjusted for differences in body composition and size. Our review revealed overlapping prevalence of low BMD in RLS and RRS, with a generally higher prevalence of low BMD in RLS overall compared to RRS (Table 1 and Fig. 1). Data from both low-income countries such as Uganda [45^▪^], Nigeria [47^▪^], India [46], Indonesia [48] and middle-income countries (South Africa [49], Brazil [27], Turkey [50], China [51,52], Israel [53], and Thailand [54]) as well as mixed settings (South Africa, India, Thailand, Malaysia, and Argentina [55]) show varying levels of low BMD with some studies reporting a high prevalence of low BMD in HIV-positive individuals of up to 85% [53]. However, a few authors such as Hamill *et al.*[49] from South Africa have reported comparable BMD levels between HIV-infected women and appropriate uninfected controls regardless of disease severity. The high BMI of participants in this study may have had a sparing effect on bone loss. In contrast, a study comparing ART-naïve to ART-experienced patients on long-term suppressive ART in western India found extremely high prevalence of low BMD, 80.4% among ART-experienced, and 67% among ART-naïve patients, but no local uninfected controls were used [46]. Another cross-sectional study among young HIV-infected Israeli women of Ethiopian and Caucasian origin found a higher prevalence of low BMD, 85% among Ethiopians compared to 40% seen in the Caucasians [53] which the authors attributed to poorer vitamin D status among Ethiopian women [53]. Similar proportions of low BMD have been reported by recently published data from RRS [56^▪^,^57^–^60^] with the exception of a few studies [61–63].

There are very limited data in any RLS regarding BMD longitudinal changes among HIV-infected persons. In a 48-week, multisite, second-line trial in South Africa, India, Thailand, Malaysia, and Argentina [55], HIV-infected patients who initiated a second-line regimen experienced additional bone loss. We did not find any longitudinal data on the effect of ART initiation on BMD among ART-naïve cohorts, or any data on fractures among HIV-infected individuals in RLS. 

**Box 1:**
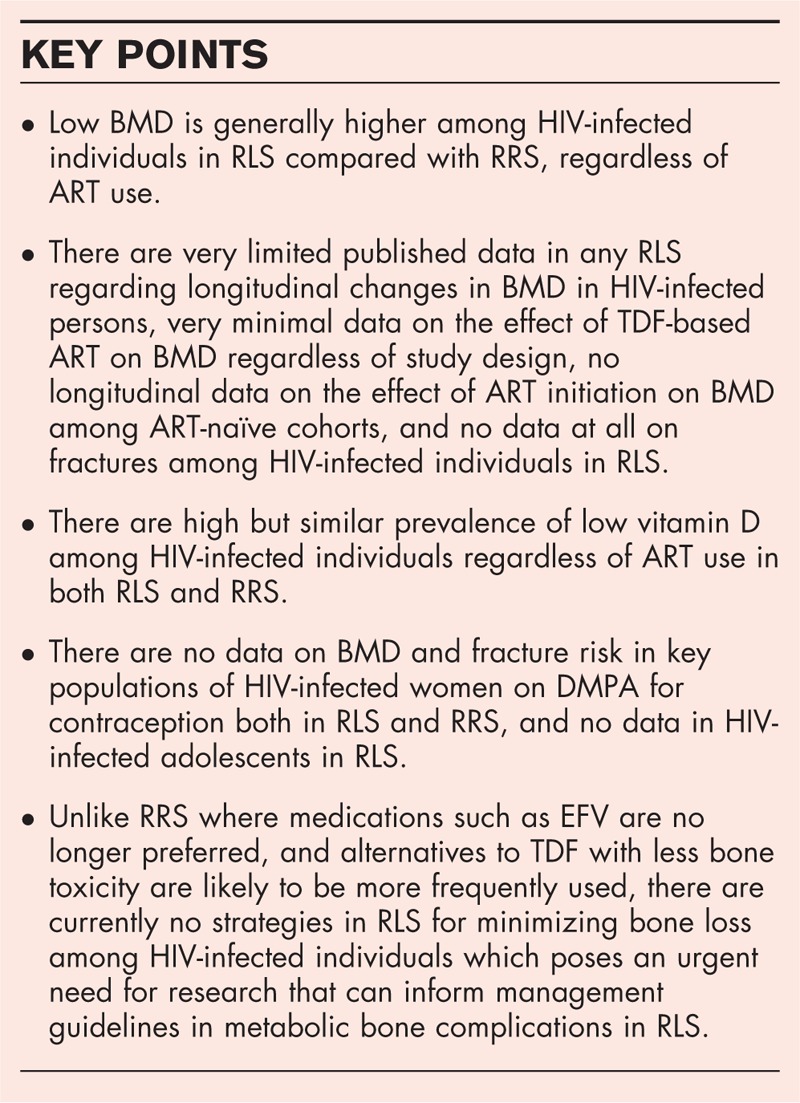
no caption available

### Role of tenofovir

A strong body of evidence from longitudinal data in RRS shows that among the different antiretroviral drugs, the potential effect of TDF on bone health is particularly concerning [64–70]. In ART-naïve HIV-positive individuals, initiating TDF-containing ART was associated with greater bone loss over the first few years compared to TDF-sparing regimens [67,69–71]. With ART-initiation, there is a rapid acceleration of bone turnover; bone resorption outstrips bone formation, likely accounting for the decrease in BMD [72,73]. Consistent with these findings, Brown and others [66,67,74] have shown that ART initiation is associated with a 2–6% loss of BMD over the first 48–96 weeks of therapy that does not return to baseline after prolonged HIV RNA suppression and also reoccurs after reinitiation of ART after treatment failure. In another adult study comparing TDF-containing and noncontaining regimens, Gallant *et al.*[64] observed increased bone resorption and loss in the TDF-containing arm compared to patients receiving an alternate NRTI (stavudine), at both the LS (−3.3 vs. −2.0%) and hip (−3.2 vs. −1.8%). Importantly, the majority of BMD loss was observed within the first 24–48 weeks of treatment, and thereafter, BMD loss slowed, but BMD did not recover over the 144 weeks of the study. Similarly, a study comparing TDF to abacavir an NRTI revealed a greater loss of BMD at total hip (−3.6 vs. −1.9%) and LS (−2.4 vs. −1.6%) in the TDF group. Again, BMD loss occurred closer to initiation of therapy and was maximal in the spine at 24 weeks and in the hip at 48 weeks [66]. More interestingly, switching from a TDF-containing regimen to an alternative NRTI leads to an increase in BMD [71]. Though the mechanism through which TDF reduces bone mass is not clear, there is more evidence suggesting that TDF induces renal dysfunction [75–87]. TDF has been shown to induce proximal renal tubular dysfunction that results in excessive glomerular filtration, renal tubular acidosis phosphate loss [83] and possible impairment in vitamin D hydroxylation [75,76,80,86–94].

In RLS, the two WHO recommended first line ART treatment regimens for adults and children above 15 years contain TDF; TDF, lamivudine (3TC) and EFV or TDF, emtricitabine (FTC), and EFV, which exposes many HIV-infected individuals to the negative impact of TDF on bone health [3,95]. Conversely, there are scarce data on the effect of TDF-based ART on BMD in these settings. Martin *et al.*[55] reported that HIV-infected patients who initiated a second-line regimen had a greater bone loss if they were on TDF for longer duration during the 48 weeks of the study. For every 1 year of TDF use, the femur BMD reduced by 1.58% and spine BMD by 1.65% (*P* < 0.001).

### Vitamin D and bone health in HIV

**FIGURE 2:**
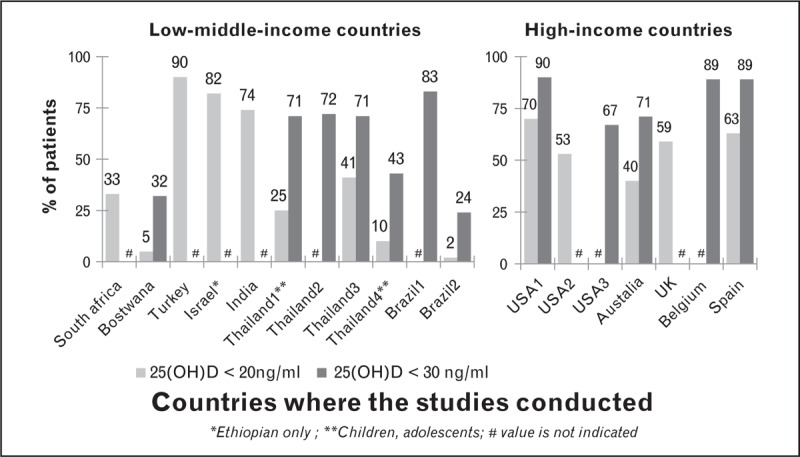
Proportion of antiretroviral-treated HIV infected patients with low vitamin D. The prevalence of low vitamin D among HIV infected individuals in both low-/low- to middle-income countries and high-income countries varies widely across studies, regardless of ART use, with insufficient levels of up to 90% in Turkey, the USA, Belgium, and Spain.

Worldwide, it's estimated that more than one billion people are characterized as having vitamin D deficiency (<20 ng/ml), or insufficiency (<30 ng/ml) regardless of the economic setting. According to a recent review by Mansueto *et al.*[96] the prevalence of vitamin D deficiency among HIV-infected individuals in both RLS and RRS varies widely across studies ranging from 25 to 93%, with an overall prevalence of 70.3 to 83.7%. Similarly, our review yielded high but similar prevalence of low vitamin D among HIV individuals regardless of ART use in both RLS [50,53,97–102] and RRS [61,103–109] with insufficient levels of up to 90% in Turkey [50] and the USA [103], Belgium [108], Spain [109] (Table 2 and Fig. 2). The authors ascribed the high prevalence of vitamin D deficiency seen among Turkish [50], and Israeli [53] to skin coverage with resultant reduced sunlight exposure. Among individuals on ART, several cross-sectional studies from both RRS and RLS have shown an association between EFV use and low 25-hydroxyvitamin D (25(OH)D) [30,97,104,110–113]. NNRTIs, especially EFV which are widely used to treat HIV infection in RLS are hypothesized to enhance 25(OH)D catabolism through the induction of cytochrome P450 enzymes (CYP24A) [112] which reduce 25(OH)D concentrations. Among HIV-infected individuals, vitamin D insufficiency has been associated with a higher risk of HIV disease progression, death and virologic failure after ART [96,114]. In addition, vitamin D deficiency has been reported to independently increase the risk of low BMD [115]. In view of this, supplementation with vitamin D has been reported to mitigate bone loss [61,105]. In a recent randomized trial Overton *et al.*[61] found that BMD loss in the first year after ART initiation may be minimized by calcium and vitamin D supplementation D. By way of contrast, none of the studies we reviewed supported an association between vitamin D insufficiency and low BMD [61]. Though a cross-sectional study by Shahar *et al.*[53] among HIV-infected Israeli women of Ethiopian and Caucasian origin reported lower levels of BMD among vitamin D deficient individuals, there findings were limited by the small sample size in addition to lack of an HIV-uninfected control group. Larger studies with a suitable comparison of HIV-uninfected controls are needed to quantify the association between vitamin D status and BMD or fracture risk in HIV populations in RLS, and whether vitamin D supplementation mitigates bone loss.

### Bone health among HIV-infected young adult women

In RLS, the HIV burden among young adult women is high [2]. Women account for approximately 57% of the 34 million people living with HIV/AIDS. Most women living with HIV are of reproductive age [2], and the provision of reproductive health services is a crucial part of their HIV care. However, certain types of hormonal contraception have been associated with long-term metabolic dysregulation, particularly low BMD. In RLS with the highest unmet need for contraception, depot medroxyprogesterone acetate (DMPA) is the preferred contraceptive option across the different age groups [116] with approximately 15 million current users in the sub-Saharan African region alone [117]. Among HIV-infected women in particular, DMPA remains effective [118] because of its lack of interactions with antiretroviral drugs [119–121]. However, owing to its hypoestrogenemic effects [122], DMPA has also been associated with reduced BMD [123–130]. The few published observational studies on the association between DMPA and fracture risk in RRS suggest increased risk of fractures among DMPA users [131,132]. For example, a large population-based control study by Meier *et al.*[131] showed a 50% increased risk of incident fractures among 20 to 44-year-old European DMPA users receiving 10 or more injections compared to nonhormonal users, among those who had received 10 or more injections. It must also be noted that all the above studies were conducted among HIV-negative individuals. Our review did not yield any published data on the effect of DMPA on BMD or fracture risk among HIV-infected women either in RRS or RLS. This presents a critical gap in policy and clinical management guidelines for HIV infected women.

### Bone health among HIV-infected adolescents

With the scale up of ART, more HIV-infected children are surviving into adolescence. In 2012, an estimated 2.1 million adolescents (10–19 years) were living with HIV in RLS [2,133], constituting over 95% of all HIV infections in this age group [2]. Although global data on ART coverage for adolescents are not available, the WHO ‘Early Release Guideline’ recommending initiation of ART in all individuals living with HIV, regardless of CD4 cell count raises further the number of adolescents in need of treatment. Perinatally infected individuals have the greatest cumulative life-time exposure to HIV and its treatment which results in increased risk of associated comorbidities, including possible reduced bone mass at a critical time of peak bone mass (PBM) accrual. Data show that a lower PBM in the young is a major determinant of subsequent osteoporosis and fracture in older adults [134–137]. Several studies from RRS support an independent, dose–response relationship between BMD and risk of osteoporotic fractures [135,138–148]. For example, a 10% increase in PBM in young women is associated with an estimated 50% reduction in fracture risk after menopause [135]. Although there have been a few controversies among HIV individuals on ART [133,149], the general conclusion from a number of studies in RRS is that TDF treatment decreases BMD with stronger associations being seen in children and adolescents than in adults [64,150–152]. Thus, BMD may be more affected during the active period of bone growth and development. Among HIV-infected adolescents living in RLS, additional highly prevalent factors, including protein and energy malnutrition, micronutrient deficiencies, and childhood infections that are known to adversely affect bone mass accrual may pose additional threats to bone acquisition. To date, there are currently no published data in RLS where over 90% of infected adolescents live. This has inadvertently lead to lack of prevention and clinical management guidelines for this unique age group who may be at considerable risk of bone complications during a critical period of PBM attainment and subsequent lifelong ART exposure.

### Constraints to diagnosis and management of bone loss in resource-limited settings

In 2015, 11 out of the 16 million people receiving ART globally were in the WHO Africa region alone [153]. However, unlike RRS where medications such as EFV are no longer preferred, and alternatives to TDF with less bone toxicity are likely to be more frequently used, there are currently no strategies in RLS for minimizing bone loss among HIV-infected individuals. The already limited funding, poor healthcare infrastructures, and sparse personnel pose tremendous challenges toward prevention and management of metabolic bone complications in RLS. As the standard assessment tool for BMD, DXA has only limited value as a single assessment. Serial assessments during HIV patient monitoring while on ART provide more information on the pattern of BMD changes [154]. In RLS, use of DXA scans in assessing BMD is limited by availability, cost, and training. In addition, once the diagnosis is obtained, the current cost of treatment medications for osteoporosis, for example, bisphosphonates is prohibitive. Furthermore, most healthcare personnel in most RLS lack the expertise to make appropriate diagnoses and provide relevant care.

### Research needs

With more people starting ART [153] and living longer with HIV than ever before, more individuals will continue to experience osteoporosis and its sequelae, including fragility fractures [155]. Given low clinical and research capacity for metabolic bone disease in RLS, there is urgent special need for building capacity in bone healthcare and research. Expanding knowledge about bone health in RLS will not only provide significant insights into the burden of HIV-related bone loss in RLS but also predictors, and evolution of bone metabolic comorbidities in the time course of HIV infection and its lifelong treatment. An initial focus is needed to establish the epidemiology of metabolic bone diseases in both the general and HIV populations. We recommend prioritization of the following research agenda in RLS:

1. Cost-effective and feasible strategies to prevent osteoporosis for both HIV-infected and noninfected populations.
2. Identification of simple-low cost tools to detect early osteopenia.
3. Strategies to minimize or avoid ARV-associated bone loss such as ART choice, dose optimization, and ARV switching.
4. Research among HIV-infected populations focusing on women of reproductive age and special populations such as perinatally infected children and adolescents.

To successfully conduct research addressing the above mentioned gaps in bone health comorbidities in RLS, there is need to work through several existing research networks either regionally or globally. This will ensure effective design and quality implementation approaches are employed. Importantly, involving key policy makers both domestically and regionally upfront will make the future policy implementation more successful.

## CONCLUSION

The review reveals overlapping prevalence of low BMD in RLS and RRS, with a generally higher prevalence of low BMD in RLS overall compared to RRS. We highlight important gaps in our knowledge about HIV-associated bone health comorbidities in RLS. In particular, there are scarce data on bone health mainly from cross-sectional studies that call for urgent need for research that can inform management guidelines in metabolic bone complications in RLS.

## Acknowledgements

F.K.M. would like to thank Professors Todd T Brown and Mary Glenn Fowler, Dr Francis Kiweewa, MU-JHU Research Collaboration, Consortium for Advanced Research and Training in Africa, Makerere University School of Public Health and University of the Witwatersrand.

### Financial support and sponsorship

F.K.M. has received an R01 grant from the National Institute of Allergy and Infectious Diseases of the National Institutes of Health (NIH) under Award Number R01AI118332NIH for bone health-related work as the Principal Investigator, and support as a site investigator on NIH funded microbicide trials network protocols. K.R. has received support from Senior Research Scholar, Thailand Research Fund (TRF) for his work.

### Conflicts of interest

There are no conflicts of interest.

## REFERENCES AND RECOMMENDED READING

Papers of particular interest, published within the annual period of review, have been highlighted as:

▪ of special interest
▪▪ of outstanding interest

## Supplementary Material

Supplemental Digital Content

## Figures and Tables

**Table 1:** Prevalence of low bone mineral density, osteopenia and osteoporosis in HIV-infected individuals in low–middle-income countries versus high-income countries

						Prevalence^*^ of			
Country (region)	Reference	Type of study	Patients	% Women	Age, mean or median (SD or IQR)	Low BMD	Osteopenia	Osteoporosis	Key findings or Remarks (DXA machine)
Low–middle-income countries (LMICs)									
Uganda (SSA)	Wandera *et al.*, 21st CROI (EARNEST trial) [45^▪^]	Cross-sectional analysis of prospective study, 2010–2014	181 HIV-infected patients failing their first line ART (duration of first line ART 3.7 (2.7–5) years, 16.7% had used TDF, CD4 67 cells/μl (35–151)	69 (12.8% postmenopausal)	35 (31, 41)	LS 50.9%, TH 24.8%	LS 42.9%, TH 23.7%	LS 8%, TH 1.1%	Low BMD at LS was associated with both low BMI and use of TDF in first-line regimen. At TH, a low BMI was predictive of low BMD. (Discovery Hologic.)
India (SA)	Dravid *et al*. [46]	Cross-sectional study, June to December 2013	536 patients:	34	42				Age, low BMI, current smoking, and menopause were associated with low BMD. Choice of ART use (TDF vs. non-TDF, PI vs. no PI) did not influence loss of BMD. (Lunar Prodigy.)
			496 HIV-infected patients on ART			LS or TH 80.4%	LS or TH 51%	LS or TH 29.4%	
			40 HIV-infected ART-naïve patients			LS or TH 67%	LS or TH 47.2%	LS or TH 19.8%	
Nigeria (SSA)	Alonge *et al.* [47^▪^]	Cross-sectional study, September to December 2010	1005 HIV-positive patients (78.1% on ART; PI 12.6%), median CD4 371 cells/μl, median VL 200 copies/ml	72	41.3 ± 10	n/a	Lt. distal radius 46.6%	Lt. distal radius 31.9%	Osteoporosis was higher in those aged >40 years, women, single, and underweight. There was no difference in BMD of those with or without PI containing medications and treatment-naïve patients. (Specific DXA machine was not indicated.)
Indonesia (EA/P)	Masyeni *et al.* [48]	Cross-sectional study, January to June 2012	73 HIV-positive ART-naïve patients (mean CD4 144.7 cells/μl and mean VL 272.3 copies/ml	32.9	33.1 ± 8.3	LS or FN 43.8%	LS or FN 35.6%	LS or FN 8.2%	Low BMD was correlated with HIV stage (*r* = 0.337; *P* < 0.001) (Lunar DPX.)
South Africa (SSA)	Hamill *et al.* [49]	Cross-sectional study, February 2010 to July 2010	75 HIV-infected ART-naïve patients with low CD4 counts (200 cells/μl)	100	33.4 ± 6.5	No significant differences in BMD at LS, TH, or FN. Proportions with osteopenia or osteoporosis were not reported.			HIV-positive women did not have lower BMD compared to HIV-negative controls, despite the pre-ARV group being lighter with lower BMI. (Hologic QDR 4500A.)
			74 HIV-positive ART-naïve with relatively preserved CD4 cell counts (>350 cells/μl)	100	33.5 ± 6.1				
			98 healthy controls	100	30.0 ± 8.1				
Turkey (EU/CA)	Aydin *et al.* [50]	Cross-sectional study, June 2010 to May 2011	126 HIV-infected patients (63.5% on ART; either AZT/3TC or TDF/FTC, LPV/r or EFV), mean CD4 313.8 cells/μl	16	40.1 ± 11.3	LS or TH 77.7%	LS or TH 53.9%	LS or TH 23.8%	Neither NNRTI nor PI containing regimens was associated with low BMD. High VL, using and duration of ART were associated with bone loss. (Norland)
China (EA/P)	Zhang *et al.* [51]	Prospective study, April 2007 to March 2011	40 HIV-infected ART-naïve patients	12.5	37.3 ± 9.9	At baseline, LS BMD of HIV-infected patients was lower than controls (1.195 ± 0.139) vs (1.138 ± 0.112). Proportions with osteopenia or osteoporosis were not reported.			With ART initiation, LS, FN, and TH BMD reduced significantly in HIV-infected patients (annual percentage decline 1.78–3.28%). (Lunar Prodigy Advance PA + 300388.)
			40 healthy controls	14.3	37.2 ± 10.3				
China (EA/P)	Wang *et al.* [52]	Cross-sectional study, January 2010 to May 2014	21 ART-naïve patients with acute HIV infection (mean CD4 420 ± 152 cells/μl, MSM 95.2%)	0	31.1 ± 6.9	LS or TH 33.3%	LS or TH 33.3%	LS or TH 0%	TH and FN BMD in patients with chronic HIV infection were lower than the other 2 groups. HIV infection, older age, lower BMI and MSM were associated with low BMD. (MEDI LINK Osteocore.)
			55 ART-naïve patients with chronic HIV infection (mean CD4 286 ± 168 cells/μl, MSM 81.7%)	0	31.6 ± 5.9	LS or TH 63.4%	LS or TH 56.3%	LS or TH 7.1%	
			71 healthy controls	0	33.7 ± 5.7	LS or TH 45.1%	LS or TH 38%	LS or TH 7.1%	
Israel (ME/NA) (Ethiopian and Caucasian origin)	Shahar *et al.* [53]	Cross-sectional study, Summer 2009	43 HIV-infected Ethiopians (mean CD4 233 cells/μl, 82% on ART, 20 study participants on PI)	100	35.9 ± 8.2	LS 85%, TH 55%, FN 65%	n/a	n/a	Low BMD in HIV-infected patients was associated with duration of HIV infection and ART use. (Lexxos, France.)
			32 HIV-infected Caucasians (mean CD4 264 cells/μl, 64% on ART, 21 study participants on PI)	100	34.8 ± 8.7	LS 40%, TH 13.3%, FN 39.3%			
Thailand (EA/P)	Wattanachanya *et al*. [54]	Cross-sectional analysis of prospective study, 2010–2011	220 HIV-positive ART-naïve patients (mean CD4 348 cells/μl)	46.8	Male 39 ± 6; female 39 ± 4	n/a	LS, TH or FN 20.9% (men), 23% (women)	LS, TH or FN 0% (men), 2.3% (women)	No difference in BMD was found between HIV-positive patients and controls (Hologic QDR4500.)
			233 healthy controls	52.4	Male 40 ± 6; female 41 ± 5	n/a	16.9% (men), 26.7% (women)	0.7% (men), 0.8% (women)	
South Africa, India, Thailand, Malaysia, Argentina	Martin *et al*. [12]; Haskelberg*et al*. (second-line trial) [55]	Prospective study, 2010–2011	210 HIV-infected patients failing their first line ART with current CD4 202 (104–307) cells/μl; median duration of ART use 3.4 years, AZT 34%, d4T 48%, TDF 17% at baseline	52	38.8 (32.9–44.2)	n/a	LS 31.3%, TH 19.7% (at baseline)	LS 5%, TH 1.5% (at baseline)	Reduced BMD was associated with longer duration of TDF and low BMI. An NRTI-sparing ARV regimen of LPV/r and raltegravir is associated with less bone loss than a LPV/r regimen containing NRTIs. BMD decrease was greatest at 48 weeks with stabilization to week 96, but no recovery. (Lunar-India, Malaysia, Argentina, Thailand or Hologic-Thailand, South Africa.)
High-income countries (HICs)									
USA1 (NA)	Battalora *et al.* (HOPS and SUN study) [56^▪^]	Cross-sectional analysis of prospective study, 2004–2012	1006 HIV-infected patients with median CD4^+^ 461 cells/μl (96.6% on ART, 67% non-Hispanic White)	17	43 (36, 49)	FN 40%	FN 36%	FN 4%	During 4,068 person-years of observation, 85 incident fractures occurred, predominantly rib/sternum, hand, foot, and wrist. Osteoporosis and current/prior tobacco use were associated with incident fracture. (Lunar or Hologic, reference standard: NHANES III database.)
UK^a^ (EU/CA)	Short *et al.* [57]	Cross-sectional study, May to August 2008	168 HIV-infected patients (63% on ART; PI 27%, NNRTI 45%, NRTI included TDF 47%)	0	45 (38, 51)	n/a	LS, TH, or FN 58%	LS, TH, or FN 12%	Number of fractures since HIV diagnosis was increased among those with osteoporosis. Duration of infection >13 years was associated with osteoporosis. (Hologic QDR4500C.)
Italy (EU/CA)	Mazzotta *et al.* [58]	Cross-sectional study, April 2009 to March 2011	163 HIV-infected patients (79.7% on ART; PIs 59.2%, TDF 70%)	29.4	44.2 ± 10	LS or TH; 63.2%	LS or TH; 49.7%	LS or TH; 13.5%; LS or TH 19.6% (Z-score ≤ −2)	Low BMD was associated with lower BMI, AIDS diagnosis, HCV co-infection, ART, and nontraumatic fractures (NTBFs). Prevalence of NTBFs was 27.0%, predictors; male sex, HCV co-infection, lower FN Z-scores. (Hologic QDR 4500A.)
The Nether-lands (EU/CA)	Kooij *et al*. [59]	Cross-sectional study, 2010–2012	581 HIV-positive patients (94.7% on ART; NRTI/TDF 96.4/77.1%, PI 43.6%, NNRTI/NVP 60.4/ 30.2%)	11.5	52.7 (48.3, 59.4)	n/a	LS 34%, FN 43%, TH 29%	LS 11%, FN 4%, TH 2%	Low BW was negatively associated with BMD in HIV-positive persons. Regardless of HIV status, younger MSM had lower BMD than older MSM, heterosexual men, and women. (Hologic QDR 4500 W, the reference standard: NHANES database.)
			520 HIV-negative controls	15.2	52.0 (47.9, 58.0)	n/a	LS 35%, FN 34%, TH 16%	LS 6%, FN 1%, TH 0%	
Japan (EA/P)	Kinai *et al*. [60]	Cross-sectional study, February 2012 to June 2013	184 HIV-infected men (93% on ART; PIs 64%, TDF 62%), median CD4 493 cells/μl	0	43 (38, 51)	n/a	LS 46% FN 54%	LS 10% FN 12%	Low BMD was associated with long-term treatment with a PI and a low BMI. Patients who discontinued PI had a higher BMD than those who currently use PI at LS but not at FN. (Hologic QDR 4500 W.)
USA2^b^ (NA)	Overton *et al*. [61]	Cross-sectional analysis of prospective study, September 2011 to February 2012	165 HIV-infected patients on EFV/FTC/TDF regimen (33% non-Hispanic Black; median CD4 341 cells/μl)	9.7		At baseline			Authors evaluated vitamin D3 (4000 IU daily) plus calcium (1000 mg calcium carbonate daily) supplementation on bone loss associated with ART initiation. BMD loss in the first year after ART initiation may be minimized by calcium and vitamin D supplementation. (Specific DXA machine was not indicated.)
			Pre vitamin D/Calcium group (*n* = 79)		36 (28, 47)	LS 9%; TH 5%	n/a	n/a	
			Placebo group (*n* = 86)		31 (25, 44)	LS 10%; TH 6%			
USA3^c^ (NA)	Cotter *et al*. [62]	Cross-sectional analysis of prospective study February 2011 to July 2012	210 HIV-positive patients (40% African)	41	39 (33, 46)	At baseline; LS 24.3%; TH 13.8%; FN 23.8%	n/a	n/a	HIV was independently associated with lower BMD at femoral neck, total hip and lumbar spine. Lunar Prodigy DXA (GE Medical Systems, Madison, Wisconsin, USA.)
			264 HIV-negative controls (25% African)	56	42 (34, 49)	LS 12.5%; TH 5.7%; FN 11.7%	n/a	n/a	
Mixed LMICs and HICs									
Australia, Belgium, Brazil, India, Ireland, Peru, South Africa, Spain, Thailand, UK, US	Carr *et al*. (STARTBone Mineral Density Substudy) [63]	Cross-sectional analysis of prospective study, June 2011 to June 2013	424 ART-naïve participants with mean CD4 688 ± 152 cells/μl	26	34 ± 10.1	LS, TH or FN 35.1%, FN 18.8%	n/a	LS, TH or FN 1.9%, FN 0.5%	Lower BMD was associated with female sex, Latin/ Hispanic ethnicity, lower BMI and higher estimated GFR. Longer time since HIV diagnosis was associated with lower TH BMD, but not with CD4 cell count or viral load. [Lunar or Hologic, reference standard: NHANES III database (hip) and Hologic's reference data (spine).]

Data shown in the table include published articles and abstracts related to prevalence of low BMD in HIV-infected adolescents or adults from RLS in 2014 and 2015 plus articles of special interest from 2013. However, for RRS, only the articles published in mid-2014 to 2015 and had more than 100 HIV-infected participants were included. BMD, bone mineral density; FN, femoral neck; HICs, high-income countries; LMICs, low–middle-income countries; LS, lumbar spine; n/a, not available; TH, total hip; VL, viral load.

^*^BMD was assessed by DXA either central or peripheral sites. In most studies, low BMD, osteopenia, and osteoporosis were defined as T-score < −1, T-score between −2.5 and −1, and T-score less than −2.5, respectively.

^a^In this study, low BMD was defined as Z-score < −2 at LS or TH.

^b^In this study, osteopenia and osteoporosis were defined as T- or Z-score < −1 and T- or Z-score < −2.5, respectively.

^c^In this study, low BMD was defined as T-score <1 in those older than 40 years or Z <2.0 in those younger than 40 years, respectively.

**Table 2:** Prevalence of low vitamin D in HIV-infected individuals in low- to middle-income countries versus high-income countries

Country (region)	Reference	Type of study	Patients	% Women	Age (mean or median; SD, IQR)	Results	Remarks
Low–middle-income countries (LMICs)							
Turkey (EU/CA)	Aydin *et al*. [50]	Cross-sectional study, June to October 2010	96 HIV-infected patients (80.2% on ART)	18	40.1 (range, 20–70)	Patients on ART: 14.3% had 25(OH)D <10 ng/ml, 67.5% had 25(OH) D 10–20 ng/ml; patients without ART: 15.8% had 25(OH)D <10 ng/ml, 73.7% had 25(OH) D 10–20 ng/ml	25(OH)D levels were low in women with veiled dressing style. No relation between low BMD and 25(OH)D levels.
Israel (ME/NA) (Ethiopian origin and Caucasian origin)	Shahar *et al*. [53]	Cross-sectional study, Summer 2009	43 HIV-infected Ethiopians (mean CD4 233 cells/μl, 82% on ART, 20 study participants on PI)	100	35.9 ± 8.2	65% had 25(OH)D <10 ng/ml 16.6% had 25(OH)D 10–20 ng/ml	PIs used were LPV/r, invirase/r, IDV. All participants were living in Israel for at least 10 years. Significantly more Ethiopian than Caucasian women covered their face and hands.
			32 HIV-infected Caucasians (mean CD4 264 cells/μl, 64% on ART, 21 study participants on PI)	100	34.8 ± 8.7	6.25% had 25(OH)D <10 ng/ml 15.6% had 25(OH) D 10–20 ng/ml	
Batswana (SSA)	Steenhoff *et al*. [97]	Prospective study, December 2011 to April 2012	60 HIV-infected study participants (PI 25%, EFV 33%, NVP 42%, TDF/ NNRTI 13%)	50	19.5 ± 12	At baseline: 5% had 25(OH)D <20 ng/ml 26.5% had 25(OH)D 20–31 ng/ml. Mean 25(OH)D was 36.5 ng/ml in 4000 IU group and 34.5 ng/ml in 7000 IU group. At 12 weeks: 1.5% had 25(OH)D <20 ng/ml 16.8% had 25(OH)D 20–31 ng/ml. Mean 25(OH)D was 54.8 ng/ml in daily 4000 IU group and 56.5 ng/ml in daily 7000 IU group.	Δ25D was two-fold higher in study participants on EFV or NVP compared to those on PIs. At 6 weeks, both NNRTI regimens resulted in greater Δ25D than those on PIs. Study participants on TDF did not differ in Δ25D from study participants on other regimens.
Thailand1 (EA/P)	Chokephaibulkit *et al.* [98]	Cross-sectional study, October 2010 to February 2011	101 perinatally HIV-infected adolescents on ART (NNRTI-based: NVP 30%, EFV 20%, and PI-based: 50%), median CD4 646 cells/μl	50	14.3 (13, 15.7)	Median 25(OH)D was 24.8 ng/ml, 24.7% had 25(OH)D <20 ng/ml, 46.5% had 25(OH)D 20–30 ng/ml	No associations between vitamin D deficiency and BMI, BMD, EFV use, HIV RNA, CD4, or self-reported sunlight exposure were observed.
Thailand2 (EA/P)	Avihingsanon *et al.* [99]	Cross-sectional analysis of cohort, July 2010 to June 2011	673 HIV-infected adults (93% on ART; EFV 31%, TDF 79% and 57% of patients had previously used d4T), median CD4 571 cells/μl	47	41.5 (37.2, 47)	40.6% had 25(OH)D <20 ng/ml, 29.9% had 25(OH)D 20–30 ng/ml	Female sex, age >37 years, and EFV use were independent predictors of hypovitaminosis D.
Thailand3 (EA/P)	Aurpibul *et al.* [100]	Cross-sectional study, March to September 2011	80 perinatally HIV-infected children on ART (NVP-based 55%, EFV-based 31%, PI-based 14%), median CD4 784 cell/μl	56	12.2 (9.1, 14.3)	Median 25(OH)D was 33.5 ng/ml, 10% had 25(OH)D <20 ng/ml, 33% had 25(OH)D 21–29 ng/ml	Only geographic location was significantly associated with low vitamin D level.
Brazil 1 (LA)	Sales *et al*. [101]	Cross-sectional study, August 2011 to December 2013	32 HIV-infected women (most were on ART but type of ART was not indicated)	100	41.7	15.63% had 25(OH)D<10 ng/ml; 65.63% had 25(OH)D 11–29 ng/ml	Factors related to the virus itself and to the use of ART may have contributed for the low vitamin D levels.
			66 HIV-infected men	0	39	18.75% had 25(OH)D >30 ng/ml; 12.12% had 25(OH)D<10 ng/ml, 71.43% had 25(OH)D 11–29 ng/ml, 15.31% had 25(OH)D >30 ng/ml	
Brazil 2 (LA)	Canuto *et al*. [102]	Cross-sectional study, September 2013	125 HIV-infected patients (83.2% on ART but type of ART was not indicated)	51.2	40.3 ± 11	Mean 25(OH)D was 39.3 ng/ml 1.6% had 25(OH)D ≤20 ng/ml 22.4% had 25(OH)D 21–29 ng/ml	Higher 25(OH)D levels were associated with female sex, no use of sunscreen, and previous opportunistic infections. Lower values were associated with the use of ART, overweight and obesity.
High-income countries							
USA 1 (NA)	Schwartz, Moore *et al*. [103]	Cross-sectional study, October 2009 to January 2010	507 HIV-negative study participants	100	41.3 (33.6, 48.7)	Median 25(OH)D was 14 ng/ml; 72% had 25(OH)D <20 ng/ml; 18% had 25(OH)D 20–30 ng/ml; Median 25(OH)D was 14 ng/ml; 70% had 25(OH)D <20 ng/ml; 20% had 25(OH)D 20–30 ng/ml; Median 25(OH)D was 17 ng/ml; 57% had 25(OH)D <20 ng/ml; 24% had 25(OH)D 20–30 ng/ml	Vitamin D levels were lower if ART included efavirenz (15 vs. 19 ng/ml, *P* < 0.001).
			358 HIV-positive ART naive patients	100	42.9 (36.3, 49.6)		
			893 HIV-positive patients on ART (PI 61%, NRTI 98%, NNRTI 26%)	100	44.9 (39.3, 50.7)		
USA 2 (NA)	Hidron *et al*. [104]	Cross-sectional study, 2007–2010	933 HIV-infected patients (82% on ART; TDF/EFV 31.6%, TDF without EFV 29.9%, no TDF 20.6%)	2.5	50 (range, 24–86)	Median 25(OH)D was 19 ng/ml 53.2% had 25(OH)D <20 ng/ml	Risk factors for vitamin D deficiency in HIV-positive patients included black race, winter season and higher GFR, increasing age and TDF use.
			5355 HIV-negative study participants	13.1	63 (22–97)	Median 25(OH)D was 24 ng/ml, 38.5% had 25(OH)D <20 ng/ml	
USA 3 (NA)	Lake *et al*. [105]	Cross-sectional analysis of prospective study, June 2010 to April 2011	122 HIV-infected patients on ART (PI 34%, NNRTI 58%, raltegravir 17%, TDF 80%, ABC 29%), mean CD4 520 cells/μl	5	49 (41, 55)	Median 25(OH)D was 20 ng/ml, 67.2% had 25(OH)D <30 ng/ml	After 12 weeks of vitamin D supplementation (vitamin D3 50000 IU twice weekly for 5 weeks, then 2000 IU daily), 81% of insufficient persons achieved 25OHD ≥30 ng/ml. 25OHD repletion rates were comparable between HIV-positive patients and controls.
Australia (EA/P)	Klassen *et al*. [106]	Cross-sectional study, January 2008 to December 2012	997 HIV-infected patients (66% on ART; EFV 24%, NNRTI ± PI 25%, no NNRTI/PI 17%)	12	41 (32,48)	Mean 25(OH)D was 24.8 ng/ml; 40% had 25(OH)D <20 ng/ml; 71% had 25(OH)D <30 ng/ml	Men, Caucasian country of origin, summer/autumn, total cholesterol to HDL ratio >5 and HIV infection were associated with vitamin D deficiency.
		May 2009 to April 2010	3653 HIV-uninfected individuals	53	50 (39, 61)	Mean 25(OH)D was 27.6 ng/ml; 22% had 25(OH)D <20 ng/ml; 63% had 25(OH)D <30 ng/ml	
United Kingdom (EU/CA)	Gedela *et al*. [107]	Cross-sectional study, January 2008 to December 2009	253 HIV-infected ART-naive study participants (64.4% were white and 35.6% were black or other ethnicity) with median CD4 450 cells/μl	18	36 (range, 16–75)	12.6% had 25(OH)D ≤10 ng/ml; 58.5% had 25(OH)D ≤20 ng/ml	Vitamin D deficiency was common among ART naive patients, with those of nonwhite ethnicity at highest risk; no association was found with CD4 cell count, HIV viral load, and HIV clinical staging.
Belgium (EU/CA)	Theodorou *et al*. [108]	Retrospective study, December 2005 to March 2011	2044 HIV-infected study participants (73.4% on ART; EFV 15.8%, 2NRTI/NNRTI 23.2%, 2NRTI/PI 35.9%, second line 14.2%)	41.5	43 (range, 20–85)	Median 25(OH)D was 13.8 ng/ml, 32.4% had 25(OH)D <10 ng/ml, 89.2% had 25(OH)D <30 ng/ml	25(OH)D <30 ng/ml is associated with general factors (female sex, winter season) and specific factors related to HIV (duration of treatment, second line treatments with multiple and complex combinations of ART). 25(OH)D <10 ng/ml is associated with a low CD4 cell count, a higher CDC stage and EFV therapy.
Spain (EU/CA)	Bañón *et al*. [109]	Prospective study, 2012	365 HIV-infected patients (98% on ART; TDF/FTC 77%, the remaining on ABC/3TC, EFV 33%)	24	44 (range, 22–75)	At baseline: 15% had 25(OH)D <10 ng/ml; 48% had 25(OH)D 10–19.9 ng/ml; 26% had 25(OH)D 20–29.9 ng/ml	After calcidiol supplementation (oral monthly dose of 16 000 IU), 25(OH)D levels increased in comparison with nonsupplemented patients (+16.4 vs. + 3.2 ng/ml; *P* < 0.01).

Data shown in the table includes published articles and abstracts related to prevalence of hypovitaminosis D in HIV-infected adolescents or adults from RLS in 2014 and 2015 plus articles of special interest ^(+)^ from 2013. However, for RRS, only the articles published in mid-2014 to 2015 and had more than 100 HIV-infected participants were included. HICs, high-income countries; LMICs, low- to middle-income countries; SSA, sub-Saharan Africa.
